# Knowledge and Attitudes Toward Premarital Screening Programs Among Students at the University of Tripoli, Libya

**DOI:** 10.7759/cureus.64274

**Published:** 2024-07-10

**Authors:** Afaf Shebani, Ariej M Mustafa, Halla Elshwekh, Abduladim Hmmier, Inas M Alhudiri

**Affiliations:** 1 Genetic Engineering, Libyan Biotechnology Research Center, Tripoli, LBY

**Keywords:** libya, premarital screening, knowledge, hereditary diseases, consanguineous marriages, attitude

## Abstract

Background: Despite the increase in hereditary disease in Arab countries due to the high rates of consanguineous marriages, research on community awareness of premarital screening (PMS) for disease carriers is still scarce.

Aim: To investigate knowledge and attitudes toward genetic PMS programs among university students in Libya.

Methods: A cross-sectional study was conducted using a self-administered questionnaire distributed to 421 Libyan students aged 18-25 years at the University of Tripoli.

Results: Most of the participants (79%, n=316) agreed that a PMS program is important and expressed willingness to have PMS programs if they were advised to do so. Two-thirds of participants (67%, n=268) had heard of PMS programs, of whom (27.2%, n=73) heard of them from social media.

Conclusion: Most of the university students had good knowledge of PMS but poor knowledge of the hereditary disease targeted by PMS. Most of them had a positive attitude toward PMS.

## Introduction

Consanguinity is marriage between relatives of at least second-degree cousins [1]. Approximately 700 million people are in consanguineous marriages worldwide [2,3]. Consanguinity plays a role but there are other possible explanations for the high prevalence rates of recessive hereditary diseases worldwide (e.g., sickle cell anemia and thalassemia) [4]. Consanguineous marriages affect the social, emotional, psychological, and cost dimensions of the family [3,5]. Studies have shown that the highest rates of consanguineous marriages are associated with low socioeconomic levels, illiteracy, and rural residence [6-8]. Some studies have reported consanguinity in some Arab countries ranging from 33-49% in Tunisia, 29-33% in Morocco and Egypt, and 48% in Libya [1,3,9-11].

According to the World Health Organization (WHO), approximately 240 million people are carriers of hemoglobinopathies, and at least 200000 affected individuals are born annually, divided about equally between sickle cell anemia and thalassemia [12].

Genetic disorders tend to be chronic, difficult, and expensive to manage, and sometimes life-threatening. The overall cost of almost all common genetic disorders in the Arab world has been estimated to be USD 13 billion per year [13]. Premarital screening (PMS) for hereditary diseases is the golden strategy for preventing genetic disorders, congenital abnormalities, and several medical and psychosocial marital problems [13]. Programs designed to determine whether individuals carry a genetic predisposition that may produce a disease in their offspring are generally recommended in several studies [12,14,15]. Several countries in the Middle East also have started such programs, including Iran (1997), Saudi Arabia (2004), the United Arab Emirates (2011), and Oman (2018), and Cyprus was one of the first countries to carry out compulsory PMS for β-thalassemia in 1973 [16,17]. Despite the increase in hereditary diseases in Libya partly due to consanguineous marriages, research on community awareness of premarital genetic screening for disease carriers is still scarce. This poses a significant challenge to the Libyan healthcare system. While the country has made progress in healthcare development, genetic testing, and counseling services are not yet widely available. For families facing genetic conditions, genetic counseling provides crucial guidance and support. Recently, the National Center for Disease Control in Libya has created a committee for genetic testing of rare diseases, which would facilitate the initiation of a premarital genetic screening program in Libya.

In Libya, there is neither an elective nor a mandatory PMS program to detect recessive genetic disorders. The aim of this study is to determine the knowledge of students at the University of Tripoli, Libya about hereditary diseases and PMS, and their attitudes toward a national PMS program. The findings of this study would be useful in starting awareness campaigns if such a program is considered in the country. 

## Materials and methods

Participants and methods

A cross-sectional study was conducted using a self-administered (printed format) questionnaire distributed to 421 currently unmarried and not previously married, Libyan undergraduate students at the University of Tripoli. The sample size was calculated using an online tool and considering the University of Tripoli population size of 76335 with a 95% confidence interval for 80% study power. The calculated sample size for this study was 383 students. The participants were recruited from the colleges of nursing, biotechnology, veterinary medicine, dentistry, medicine, pharmacy, information technology, English language, science, economics, law, agriculture, engineering, management, and education by a random convenience sampling method. These colleges were subcategorized into humanities, basic sciences, medical sciences, and applied sciences. To ensure representation from different disciplines, the study participants were divided as evenly as possible among the four subcategories. The study was approved by the National Ethics Committee of Libya, based at the Libyan Biotechnology Research Centre, Tripoli.

The participants were provided with an information sheet about the study, and they were asked to sign informed consent if they agreed to participate willingly. The information sheet included the definition of PMS and the goals of PMS programs. The questions were of two types: tick and write the answer. The questionnaire was designed by the researchers following a review of relevant literature [17-19]. The study was conducted between the 1st of September 2019 and the end of February 2020. Questionnaires with incomplete answers were excluded (21 questionnaires), and analysis was carried out on the remaining 400 completed questionnaires. We conducted a pilot test for validation of the questionnaire. We distributed the questionnaires to 20 random students to evaluate the clarity of the questions and the ease of the answering mechanism. Modifications were made accordingly.

The questionnaire consisted of four parts. The first part covered the participant’s sociodemographic data (age, sex, college), degree of consanguinity of parents, and history of disease inheritance. The second part assessed the extent of the student’s knowledge of disease inheritance, the role of consanguinity in predisposing to hereditary diseases, the importance of PMS, and religious objections to PMS. Six questions were used in the form of "yes," "no," and "I don’t know" to assess knowledge. Correct answers were scored 1, whereas incorrect and "I don't know" answers were scored 0. The overall knowledge of each participant was defined as the sum of these scores. An overall score ≤4 was classified as poor knowledge, while a score ≥5 was considered good knowledge (i.e., ≥60% score). The third part assessed their attitudes toward a PMS program (a Likert scale with answers ranging from strongly disagree to strongly agree was used). The same method was used to assess the attitude score. An overall score ≤9 was considered a negative attitude, while a score >9 was considered a positive attitude (i.e., >60% score). The 60% cut-off for the knowledge and attitude scores corresponds to a natural breakpoint because it falls at a point where there was an evident shift in the distribution of scores in the histogram. The fourth part asked about the participant's potential response to undesirable PMS results and the possibility of deciding against that marriage. We also examined their degree of awareness and whether they intend to encourage others to undertake PMS as described in the Appendix (Table 6).

Statistical analysis

Frequencies and percentages of specific responses were calculated using SPSS, version 26 (SPSS Inc., Chicago, Illinois, USA). Chi-square (χ2) analyses were used to test correlations between categorical variables. A p-value of <0.05 was considered statistically significant.

## Results

Of the 400 students included in the study, 320 (80%) were females. The participants' ages ranged from 18 to 25 years, with an average of 22.5±2.5 years. Most of the participants (n=364, 91%) were ≤20 years old. Approximately one-third of the participants (126, 31.5%) were students in basic sciences, whereas medical sciences and humanities students each represented about one-quarter of the participants (24.5%, n=98 and 26.5%, n=106, respectively). The remainder of the participants (17.5%, n=70 ) were applied sciences students (Table 1).

**Table 1:** Sociodemographic characteristics of the participants

Characteristics	N	(%)
Sex		
Female	320	80
Male	80	20
Age group (years)		
≤20	364	91
>20	36	9
Academic year		
1	92	23.0
2	90	22.5
3	122	30.5
4	96	24.0
College basic sciences	126	31.5
Humanities	106	26.5
Applied science	70	17.5
Medical science	98	24.5

Approximately half of the participants (n=194, 48.5%) reported parental consanguinity, of whom 107 (55.1%) couples were first-degree relatives (Table 2). When the participants were asked about the presence of a personal history of hereditary diseases, none of the participants reported having sickle cell anemia, thalassemia, or G6PD deficiency, the common autosomal recessive diseases in the Mediterranean and Middle East regions. Instead, they reported multi-factorial complex disorders such as asthma, diabetes, hypertension, celiac disease, migraine, eczema, hypothyroidism, visual impairment, and stammering. Therefore, 5.8% (n=23) of the participants wrongly thought that they had hereditary diseases. Seventy-three participants (18.3%, n=73) reported Alzheimer’s disease, hypertension, diabetes, cancers, asthma, heart diseases, Down syndrome, vision loss, kidney dialysis, and Parkinson's disease as family history of inherited diseases.

**Table 2:** Parents' consanguinity and family hereditary diseases

Characteristics	N	%
Parents' consanguinity		
Yes	194	48.5
No	206	51.5
Degree of parents' consanguinity		
1st cousins	107	55.1
2nd cousins	89	45.9
Personal history of hereditary disease		
Positive	23	5.8
Negative	377	94.2
Family history of hereditary disease		
Positive	73	18.3
Negative	327	81.8

The next section of the questionnaire focused on the participants' knowledge of genes, genetic diseases, and PMS. Two-thirds of the participants (268; 67%) had heard of PMS and its importance in minimizing the penetration of both autosomal and X-linked recessive genetic diseases. Their sources of information on PMS were social media (27.2%, n=73), educational curricula (23.9%, n=64), family and friends (19%, n=51), television (14.6%, n=39), medical sources (13.8%, n=37), and other unspecified sources (1.5%, n=4).

A high percentage of the participants (85.3%, n=341) knew that genes are responsible for passing genetic diseases, whereas 14.7%, n=58, did not. Also, 85% (n=340) of the participants agreed that undertaking PMS before marriage could help in avoiding recessively inherited disorders in their offspring, while 13.3% (n=52) did not know and 1.8%, n=7.2, thought that undertaking a premarital test before marriage cannot help in avoiding inherited diseases. Only 14.2%, n=56, of the participants agreed with the statement that PMS should be undertaken only in cases of consanguineous marriages.

Most (80.3%, n=321) of the participants had good knowledge of hereditary diseases and PMS, with a mean score of 4.23±1.155. Interestingly, 91% of the participants who had heard of PMS had good knowledge (p=0.000), but there was no significant association between knowledge score and college attendance (Table 3).

**Table 3:** Participants' knowledge of PMS PMS, premarital screening

College	Poor knowledge	Good knowledge	P-value
Yes, I heard of PMS	58.3%	91%	0.000
No, I haven't heard of PMS	41.7%	9%	
Basic science	24 (19.0%)	102 (81.0%)	0.224
Humanities	22 (20.8%)	84 (79.2%)	
Applied science	19 (27.1%)	51 (72.9%)	
Medical science	14 (14.3%)	84 (85.7%)	
Total	79 (19.8%)	321 (80.3%)	

In another set of questions, 57.3% (n=129) of the participants did not prefer consanguineous marriage, while 13.5%, n=54, did. About 61.3% (n=245) of the participants did not believe that religion would be an obstacle to undertaking PMS, while 30.8% (n=123) gave a neutral response. On the other hand, 79% (n=316) of the participants agreed to carry out PMS if available, while 6.3% (n=25) stated that they would refuse to do it and 14.8% (n=59) gave neutral answers. The overall attitudes and beliefs of the participants regarding PMS are described in Table 4.

**Table 4:** The attitudes of the participants toward PMS PMS, premarital screening

Statement	Strongly agree	Agree	Neutral	Disagree	Strongly disagree
I prefer consanguineous marriage	14 (3.5%)	40 (10%)	117 (29.3%)	107 (26.8%)	122 (30.5%)
PMS doesn’t interfere with belief in destiny	93 (23.3%)	152 (38%)	123 (30.8%)	25 (6.3%)	7 (1.8%)
I will carry out PMS if available	156 (39%)	160 (40%)	59 (14.8%)	18 (4.5%)	7 (1.8%)

In the last section of the questionnaire on how they expect to decide on the results of PMS, 16.1%, n=64, of the participants reported that they would marry even if there was a risk of getting a child with a disability or disease, while 55.3%, n=221, declared that they would not, and 28.8%, n=115, had no decision. Most of the participants (61.8%, n=247) agreed that a PMS program should be obligatory, while 18.5%, n=74, did not. Over half of the participants (56.5%, n=226) believed that PMS does not invade personal privacy, while 14.8%, n=59, believed that it does (Table 5). Moreover, 31.3%, n=125, of the participants supported the adoption of a law prohibiting incompatible couples from getting married. Most of the participants (83.8%, n=335) agreed to contribute to raising awareness of the importance of PMS.

**Table 5:** Other participant responses

Question or statement	Strongly agree	Agree	Neutral	Disagree	Strongly disagree
Would decide to marry even if there is a hereditary disease risk?	29 (7.3%)	35 (8.8%)	115 (28.8%)	111 (27.8%)	110 (27.5%)
Will demand implementation of a law that prohibits incompatible marriage.	56 (14%)	69 (17.3%)	143 (35.8%)	97 (24.3%)	35 (8.8%)
PMS should be obligatory.	132 (33%)	115 (28.7%)	79 (19.8%)	50 (12.5%)	24 (6%)
Will contribute to raising awareness about the importance of PMS.	166 (41.5%)	169 (42.3%)	59 (14.8%)	5 (1.3%)	1 (0.3%)
PMS program invades personal privacy.	17 (4.3%)	42 (10.5%)	115 (28.7%)	160 (40%)	66 (16.5%)

## Discussion

It is important to know the possibility of transmitting genetically inherited diseases to one’s offspring. Genetic disorders might be caused by single-gene mutations such as congenital deafness, cystic fibrosis, sickle cell anemia, neurofibromatosis, Duchenne muscle dystrophy, and familial hypercholesterolemia, by multi-factorial conditions such as autism, diabetes, spina bifida, cancer, arthritis and Alzheimer's disease, and by chromosomal abnormalities such as in Down syndrome, fragile X-chromosome, Triple-X syndrome, Trisomy 13 and 18, and Klinefelter syndrome [20-22]. There are three patterns of genetic inheritance of diseases: autosomal, X-linked (both could be dominant or recessive), and complex inheritance [23,24]. PMS, when considered, is useful in preventing the incidence of recessive inherited disorders [25,26]. PMS is also helpful in autosomal dominant disorders when both partners are affected, thus diminishing the severity of the disease and also limiting the chance of getting a diseased child to 50% instead of 75%, when preventing the marriage of two affected parents [26-28].

However, to protect the community from genetic disorders caused by consanguinity, screening for genetic disorders before marriage has been used for seven decades [29,30]. The community’s awareness of genetic disorder inheritance and screening for such diseases might reflect the community’s compliance with any future regulations that might be put in place to minimize the overall volume of disabilities in the community. In that context, this study was designed to gain insight into the awareness of university students in Libya of hereditary diseases and PMS and their attitudes toward such screening.

Here, we surveyed 400 undergraduate university students with an average age of 20 years. Their knowledge of genetic disorders and PMS and their attitudes to and acceptance of PMS programs were assessed. However, consanguineous marriages in Libya are generally prevalent, but the prevalence varies from region to region. In large cities with mixed populations, such as Benghazi and Tripoli, consanguineous marriages represented about 46%, compared with 72% in Murzuq town in the South of Libya [27]. In other coastal cities, consanguineous marriage is still maintained within a few families. In our study, almost half of the participants (48.5%, n=194) had consanguineous parents, of whom 55.2%, (n=107) were first-degree relatives. These rates are similar to those reported among Taif University students in Saudi Arabia as well as those reported in other studies [5,19]. The high rate of consanguineous marriage and the high rate of hereditary diseases in the families observed in this study indicate a lack of awareness among these parents of the genetic transmission of diseases.

Our results showed that two-thirds of the participants (67%, n=268) had heard of PMS, 27.2% (n=73) of whom 73 (27.2%) had heard of it from social media and 39 (14.6%) from TV shows. Such sources of information might reflect the characteristics of generation Z (born during 1997-2012) of tending to exhibit dependence on the internet and electronic gadgets [30]. The academic curricula came in second place, at 23.9%.

On the one hand, most of the participants were aware of the genetic transmission of hereditary diseases. On the other hand, judged by their answers on the prevalence of hereditary diseases among their families, they exhibited a poor understanding of which diseases are targeted by PMS.

One interesting finding is that most of the participants (79%) expressed willingness to undertake PMS if it is available and they were advised to do so, and only a minority (6.3%) expressed unwillingness (Table 3). Similar findings have been reported in other Arab countries [17]. Another positive finding is that 57.3% of the participants did not prefer consanguineous marriage whereas 13.5% did. The remainder (29.3%) gave neutral answers (Table 4). This category of students might benefit from social media and TV shows to gather more information about the potential negative consequences of consanguineous marriage.

Over half of the participants (55.3%, n=221) declared that they would not marry their prospective partners if PMS showed incompatibility. This finding is similar to that of a population-based study in Saudi Arabia [30]. Notably, 61.7% (n=247) of the study participants felt it important to protect their community from more preventable disabilities by implementing obligatory PMS, which resembles the pattern observed among university students in Oman [17]. Moreover, 31.3% (n=125) of the participants tended to support legislation banning marriages that are considered incompatible in the context of inherited diseases. This may have been motivated by their awareness of the risks and consequences of having children with a hereditary disease. However, 33.1% (n=132) did not support this idea, possibly because of their belief in fate. Our study results differ from a Saudi Arabian study in which 91.8% (n=367) of the participants supported the notion of demanding the implementation of such a law [5].

Unfortunately, we were unable to gather a sufficient number of questionnaires from male participants (20%), who tended to avoid participation, did not take the matter seriously, and in most cases refused to complete the questionnaire. Cultural norms around masculinity or social stigmas related to reproductive health could be one justification for men to avoid such discussions. Despite this, the study provides valuable insights from female students, who may play a key role in discussions and decisions surrounding premarital genetic screening in Libya. Future research to specifically target male students would be crucial to gain a more comprehensive understanding of perspectives on PMS in Libya. In addition, other studies could be conducted to directly understand the reasons behind male students' reluctance to participate or their potential negative attitudes.

Self-administered questionnaires can introduce bias, and the university sample may not reflect the general population's knowledge and attitudes due to potential selection bias. Surveying couples from the general population at mandatory PMSs (it is mandatory by law in Libya for couples going to get married to test for hepatitis B and C and HIV) in future studies is suggested.

The healthcare system in Libya is evolving with new objectives being set for establishing diagnostic and preventive genetic testing. This made it important to assess the attitude and knowledge toward genetic screening. Implementing PMS programs in Libya necessitates collaboration between key stakeholders like the Ministry of Health, universities, and religious institutions. While such programs offer potential benefits, careful consideration must be given to potential risks like discrimination and privacy concerns. To mitigate these risks, standardized protocols, non-discrimination policies, pre- and post-test counseling, and robust data security measures are crucial.

## Conclusions

To the best of our knowledge, this is the first study in Libya assessing university students' knowledge of and attitudes toward PMS. The study showed that while awareness of hereditary diseases as a consequence of consanguinity is common among university students, their knowledge of the hereditary diseases targeted by PMS is deficient. Nevertheless, most of them are sufficiently aware to be able to make informed decisions in the context of a PMS program, and most of them have a positive attitude toward PMS.

## Appendices

**Table 6:** Questionnaire

General information and family history of genetic disease		
Sex	Male	Female
Age		
Address		
Marital status		
Academic year		
College		
Are you married to a relative?	YES	NO
If the answer is yes, what is the relationship of relatives?	First degree	Second degree
Are your parents relatives?	YES	NO
If the answer is yes, what is the relationship of relatives?	First degree	Second degree
Do you suffer from a hereditary disease?	YES	NO
If the answer is yes, what is the disease?		
Do you have a family history of a hereditary disease?	YES	NO
If the answer is yes, what is the disease?		

Participants’ knowledge about genetic diseases and the premarital genetic screening program						
Are genes responsible for transmitting hereditary diseases?	YES		NO		I do not know	
Have you ever heard of the premarital genetic screening program?	YES		NO			
If the answer is yes, what is the source of your knowledge about it?	Television	Social networking sites	Friends	Medical sources	Curriculum	
Do you think that the genetic screening program applies to consanguineous marriage only?	YES		NO		I do not know	
Do you think that consanguineous marriage increases the chance of genetic diseases in children?	YES		NO		I do not know	
Do you think that the genetic screening program reveals the possibility of having children?	YES		NO		I do not know	

Participants’ behavior and motivations regarding the premarital genetic screening program:					
Question	Strongly disagree	Disagree	Neutral	Agree	Strongly agree
Do you prefer consanguineous marriage?					
Early screening before marriage does not interfere with my belief in destiny					
If a genetic screening program was available before marriage, would you do it?					
If you agree, answer the following questions					
To prevent the transmission of genetic diseases to the next generation					
To ensure the health and safety of the partner					
I'll do it just so you know					
If you do not agree, answer the following questions					
I will marry my relative (partner) anyway					
For fear that the result will be unsatisfactory					

The reactions of the polytheists after knowing the result:					
Question	Strongly disagree	Disagree	Neutral	Agree	Strongly agree
I will marry my relative even if there is a risk that my children will develop a genetic disease					
I will change the marriage decision in case of incompatibility					
I will demand the implementation of the law preventing marriage in the event of incompatibility					
Genetic testing before marriage should be mandatory					
I will contribute to raising awareness about the importance of genetic testing before marriage					
